# The Effect of Lubricin on the Gliding Resistance of Mouse Intrasynovial Tendon

**DOI:** 10.1371/journal.pone.0083836

**Published:** 2013-12-13

**Authors:** Masanori Hayashi, Chunfeng Zhao, Andrew R. Thoreson, Takako Chikenji, Gregory D. Jay, Kai-Nan An, Peter C. Amadio

**Affiliations:** 1 Biomechanics Laboratory, Division of Orthopedic Research, Mayo Clinic, Rochester, Minnesota, United States of America; 2 Department of Emergency Medicine, Rhode Island Hospital and Brown University, ‪‬‬‬‬‪Providence, Rhode Island, United States of America; Van Andel Institute, United States of America

## Abstract

The purpose of this study was to investigate the role of lubricin on the gliding resistance of intrasynovial tendons by comparing lubricin knockout, heterozygous, and wild type mice. A total of thirty-six deep digital flexor (DDF) tendons in the third digits of each hind paw from eighteen adult mice were used, including six lubricin knockout mice (*Prg4* –/–), six heterozygous mice (*Prg4* +/–), and six wild type mice (*Prg4* +/+). The tendon gliding resistance was measured using a custom-made device. Tendon structural changes were evaluated by scanning electron and light microscopy. The gliding resistance of intrasynovial tendons from lubricin knockout mice was significantly higher than the gliding resistance of either wild type or heterozygous mice. The surface of the lubricin knockout tendons appeared to be rougher, compared to the wild type and heterozygous tendons. Synovial hyperplasia was found in the lubricin knockout mice. Cartilage-like tissue was found in the tendon and pulley of the lubricin knockout mice. Our findings confirm the importance of lubricin in intrasynovial tendon lubrication. This knockout model may be useful in determining the effect of lubricin on tendon healing and the response to injury.

## Introduction

Lubricin, a mucinous glycoprotein, was originally isolated from synovial fluid, and has been demonstrated to provide articular cartilage lubrication[1–3]. The lubricating properties of lubricin are similar to normal synovial fluid[4–6]. Lubricin also reduces synovial cell overgrowth and hinders integrative repair of cartilage[7–9]. Lubricin has also been found on the flexor tendon surface, and has been shown to play an important role in tendon lubrication[10–12]. In addition, recent in vitro studies have reported that lubricin decreases tendon gliding resistance[13,14]. 

Camptodactyly-arthropathy-coxa vara-pericarditis syndrome (CACP) is “an autosomal recessive disorder of precocious joint failure associated with noninflammatory synoviocyte hyperplasia and subintimal fibrosis of the synovial capsule”. CACP patients have defective lubricin synthesis[15]. Recently a mouse lacking lubricin has been created, with a phenotype similar to CACP patients, including cartilage degeneration, synoviocyte hyperplasia due to cell proliferation and interphalangeal joint contractures[7].

Little is known about intrasynovial tendon function in either CACP patients or lubricin deficient mice, which also display forepaw and hindpaw curved digits[7] similar to the camptodactyly seen clinically in patients with CACP syndrome. Recently Kohrs et al. compared intratendinous tendon fascicle gliding resistance in wild type and lubricin knockout mouse and found the absence of lubricin increased tendon fascicle gliding resistance[16]. We hypothesized that the absence of lubricin would increase gliding resistance not only between fascicles within tendons, but also on the tendon surface. The purpose of this study was therefore to investigate the role of lubricin on the surface of intrasynovial tendons by comparing gliding resistance, surface morphology, and structural histology in lubricin knockout, heterozygous, and wild type mice.

## Methods

### Specimen Preparation for Measurement of Tendon Gliding Resistance

**Figure 1:**
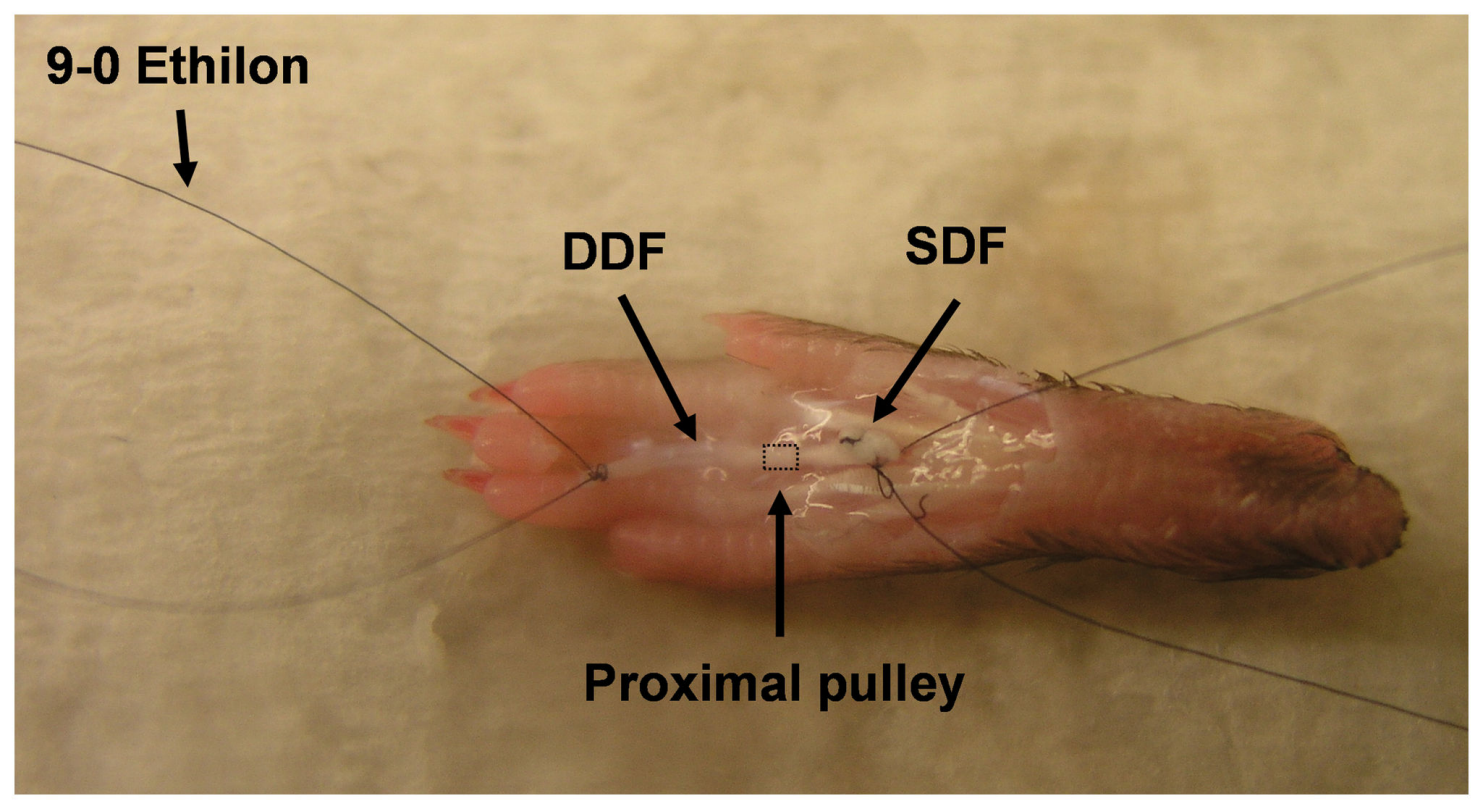
Photograph of a dissected mouse hind paw. Skin and synovial sheath have been removed from 3rd digit. DDF=deep digital flexor, SDF= superficial digital flexor.

 The details of the lubricin knockout mouse model have been described previously[7]. Thirty-six hind paws were obtained from eighteen adult mice, aged 15–17 weeks, including six lubricin knockout mice (*Prg4* –/–), six heterozygous mice (*Prg4* +/–), and six wild type mice (*Prg4* +/+). The mice were sacrificed for other projects, not involving the paws, which had been approved by the Institutional Animal Care and Use Committee (IACUC). This work was approved by the Lifespan Animal Welfare Committee at Rhode Island Hospital and Brown University. A total of thirty-six deep digital flexor (DDF) tendons in the third digits of each hind paw were used for measurement of tendon gliding resistance (n=12). The dissections of the mouse hind paws were performed as described by Wong et al.[17] A longitudinal incision was made in each paw and the flexor sheath, including the distal pulley, was removed. The tendon was marked at the proximal edge of the proximal pulley in full digit extension position. A single 9-0 Ethilon (Ethicon, Somerville, New Jersey, USA) suture loop was made at each end of the tendon, and then the tendon was cut at the outside of each loop. (Figure 1)

### Measurement of Tendon Gliding Resistance

**Figure 2:**
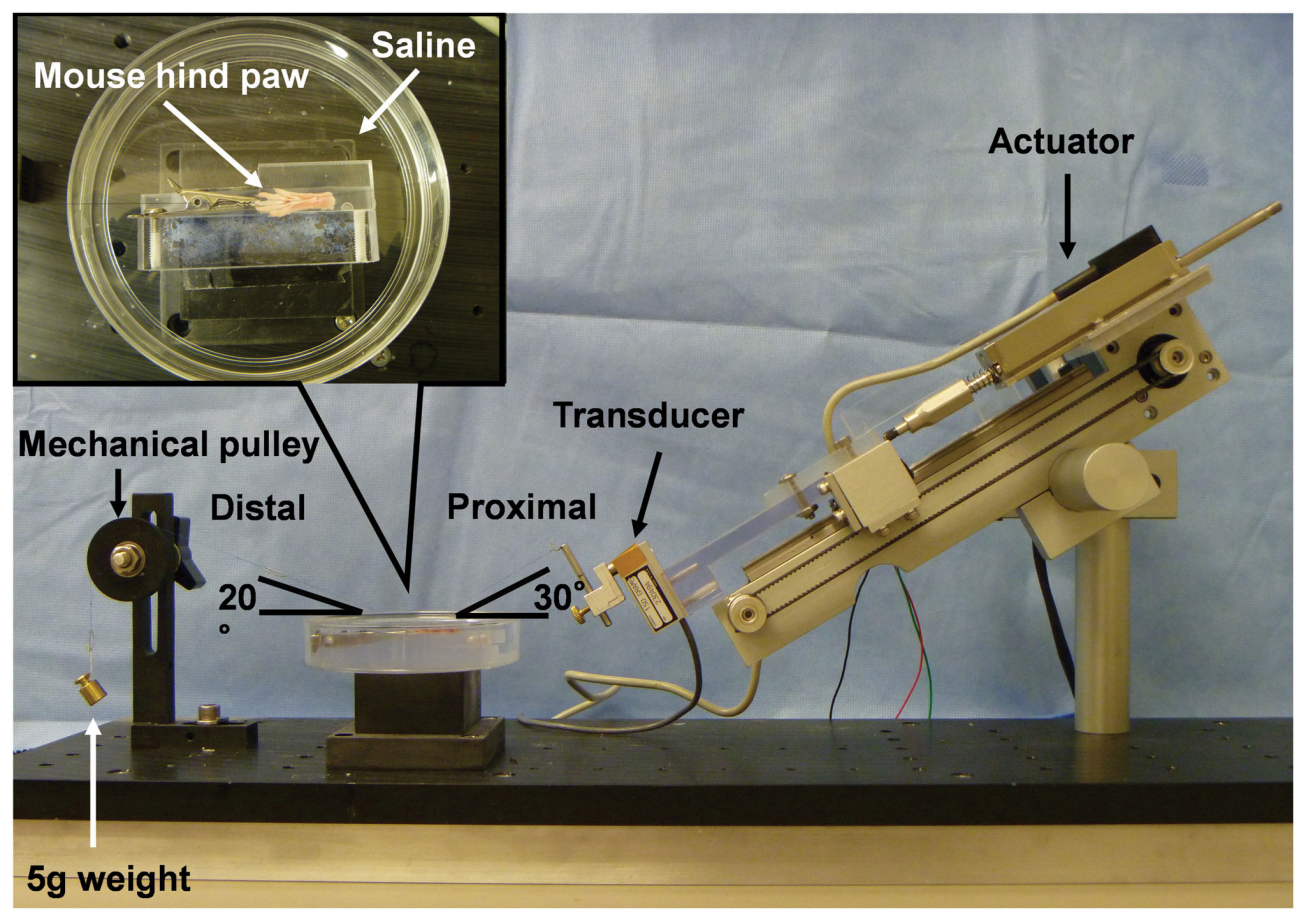
Photograph of the testing apparatus for measuring gliding resistance between the DDF tendon and its proximal pulley.

A previously described and validated testing device was modified to measure the gliding resistance between the DDF tendon and the proximal pulley[18]. (Figure 2) Each paw was fixed on a flat platform with the third digit kept in full extension in a saline bath. The dorsal side of the paw was affixed with cyanoacrylate adhesive (Loctite 401, TMs of Loctite Corp. Hartford, CT, USA) and the digit was held with a small clip. The proximal end of the DDF tendon was attached to a 150-g load transducer (GS0-150, Transducer Techniques, Temecula, CA, USA). A 5-g weight and cord was attached to the distal end of the tendon and passed over a low-friction mechanical pulley to apply a tension to the DDF tendon. The load transducer was connected to a custom linear actuator driven by a precision gear head stepper motor controlled by a motor driver/microcontroller (ACE-SDE, Arcus Technology, Livermore, CA, USA). 

An arc of contact of 50° was selected to measure the gliding resistance, a value selected based on the experience of previous studies[18]. The entire paw, including the middle digit pulley and tendon was immersed in saline during the entire test. The actuator pulled the tendon proximally at a rate of 0.25 mm/s. Maximum excursion was set at 4 mm, based on pilot testing that showed that smaller excursions only deformed the pulley but did not result in any actual tendon motion against the pulley. After two cycles of motion to precondition the material, data were collected from a third flexion/extension cycle. The force (F) at the proximal force transducer and the corresponding displacement was recorded at a sampling rate of 20 Hz. The total gliding resistance (TGR) during tendon flexion and extension was calculated based on previous publications[18]. However, in this testing setup, there was no second force transducer attached at the distal tendon end. Therefore, the TGR included not only the gliding resistance at the interface between the tendon and pulley (TP), but also the resistance of the mechanical pulley (MP). 

Since the gliding resistance was expected to be small, accurate measurement and appropriate calculation are important. In order to distinguish these two different resistance sources, the friction of the mechanical pulley (F_MP_) alone, though small, was characterized by applying similar loading conditions described above without the mouse tissue present with the same calculation method[18]. Therefore, the tendon/pulley (F_TP_) gliding resistance will be: F_TP_ = TGR – F_MP_.

### Scanning Electronic Microscopy (SEM)

From each group, three DDF tendons were prepared for analysis by scanning electron microscopy (SEM). After tendons were washed in phosphate buffered saline (PBS), they were fixed in a buffered glutaraldehyde and osmium tetroxide solution. The fixed tendons were dehydrated in graded acetone, coated with gold/palladium alloy and examined by SEM (Hitachi S-4700, Hitachi, Japan) in secondary electron mode at 3kV. The tendon surface was qualitatively assessed for its smoothness.

### Histology

 From each group, three specimens were immersed for 24hours with 4% paraformaldehyde in 0.1M phosphate buffer (PB, pH7.4) at 4°C and decalcified using decalcifying solution B (Wako, Osaka, Japan) for 7 days, washed for 4 days with several changes of 0.1M PB containing 5%, 10%, 15%, and 20% sucrose and then embedded in Tissue-Tek O.C.T. Compound (Sakura Finetek, Inc, CA, USA). The specimens were cut transversely and longitudinally into 10-μm thick serial sections using a Leica microtome (Leica Microsystems, Wetzlar, Germany). The transverse serial sections of the whole specimen were washed by distilled water (DW) for 5 minutes and stained with either hematoxylin and eosin (H&E) or Alcian blue. The stained sections were dehydrated and mounted on glass slides. The morphology and cellularity were evaluated with light microscopy.

### Statistical Analysis

The average of the gliding resistance was analyzed using one-way analysis of variance (ANOVA) followed by the Tukey post hoc test. A *P*-value of 0.05 or less indicated a significant difference between groups.

## Results

**Figure 3:**
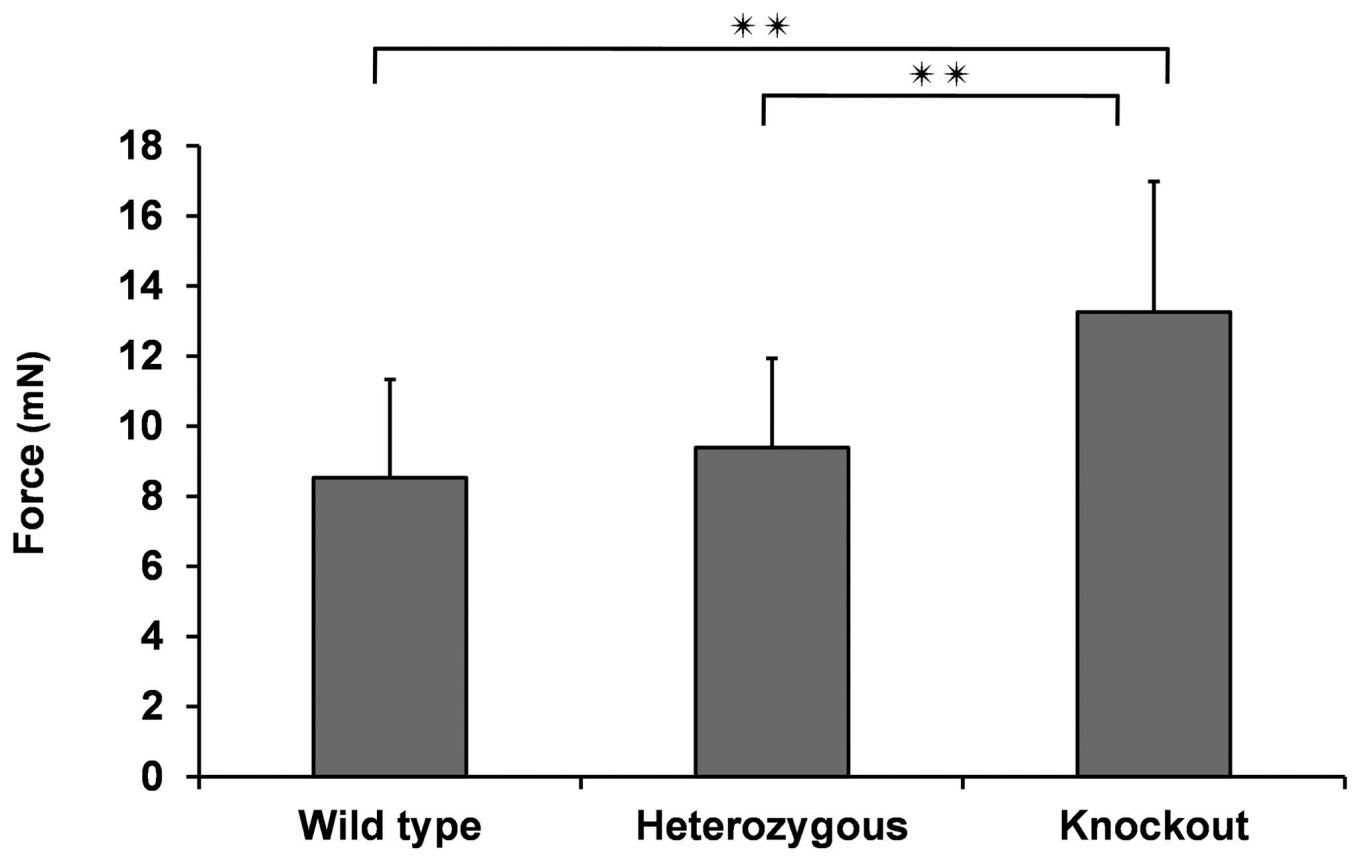
The gliding resistance between DDF tendon and proximal pulley. Results are presented as mean and standard deviation (SD) of n=12. **, P<0.01.

The gliding resistance values of the wild type, heterozygous, and lubricin knockout groups were 8.53 mN (±2.80), 9.39 mN (±2.55), and 13.3 mN (±3.73), respectively. The gliding resistance of the lubricin knockout mice was significantly higher than the gliding resistance of the wild type or heterozygous mice (p < 0.05). There was no significant difference in gliding resistance between wild type and heterozygous mice. (Figure 3)

**Figure 4:**
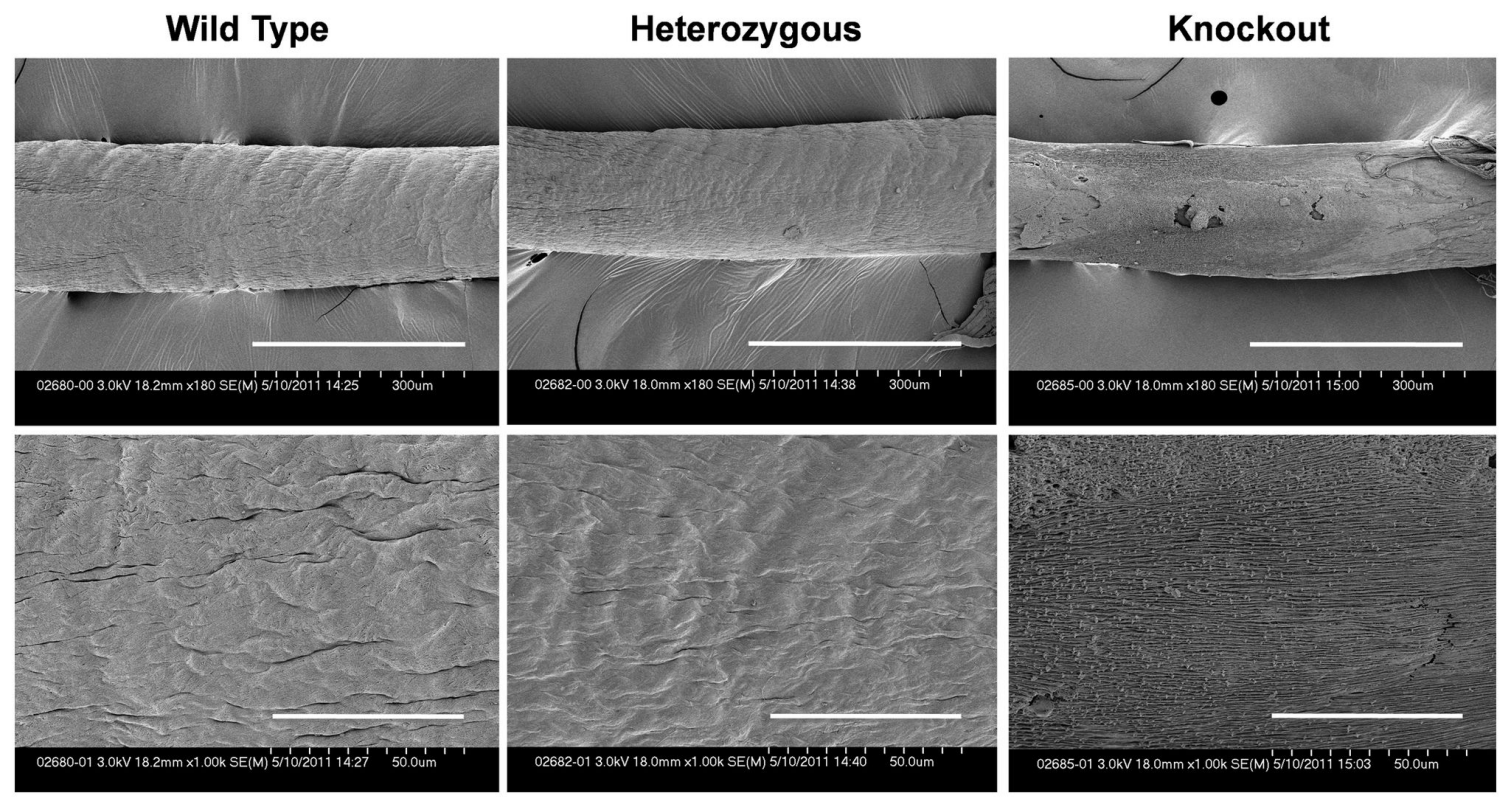
Images of the surface of DDF tendon. Upper row shows low-magnification. Bar=300 μm. Lower row shows high-magnification. Bar=50.0 μm.

 Qualitatively, on SEM the surface of the lubricin knockout tendons appeared rougher compared to the wild type and heterozygous tendons. (Figure 4) There was no obvious difference in roughness between wild type and heterozygous tendons. 

**Figure 5:**
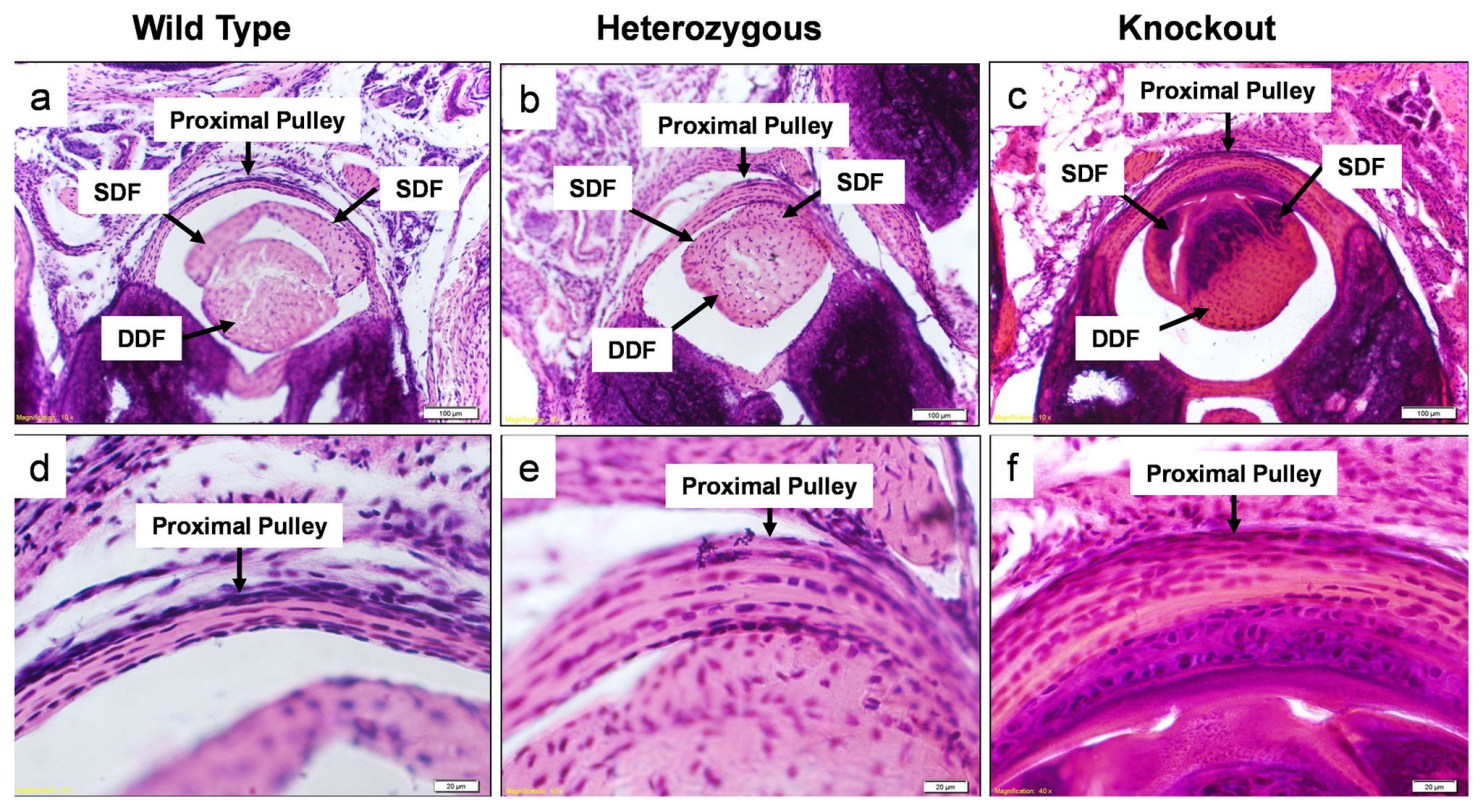
Cross sections through proximal mouse digit. Stained with hematoxylin and eosin. Upper and lower row show x100 and x400 sections respectively Note mild and more pronounced pulley thickening in the heterozygous and knockout mice, respectively, compared to the wild type.

**Figure 6:**
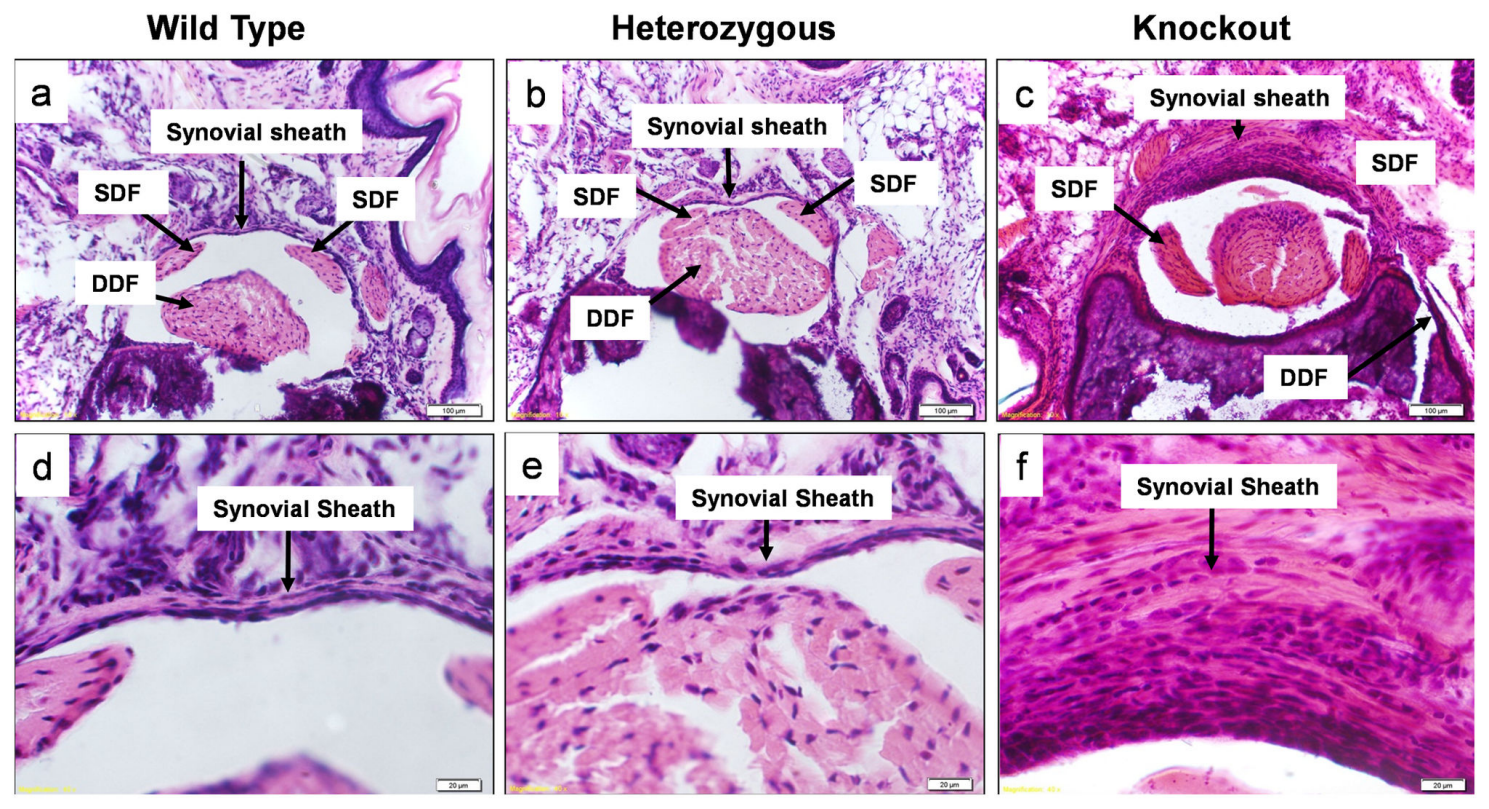
Cross sections through distal mouse digit. Stained with hematoxylin and eosin. Upper and lower row show x100 and x400 sections respectively. Note thickening of the synovial sheath in the knockout mouse.

**Figure 7:**
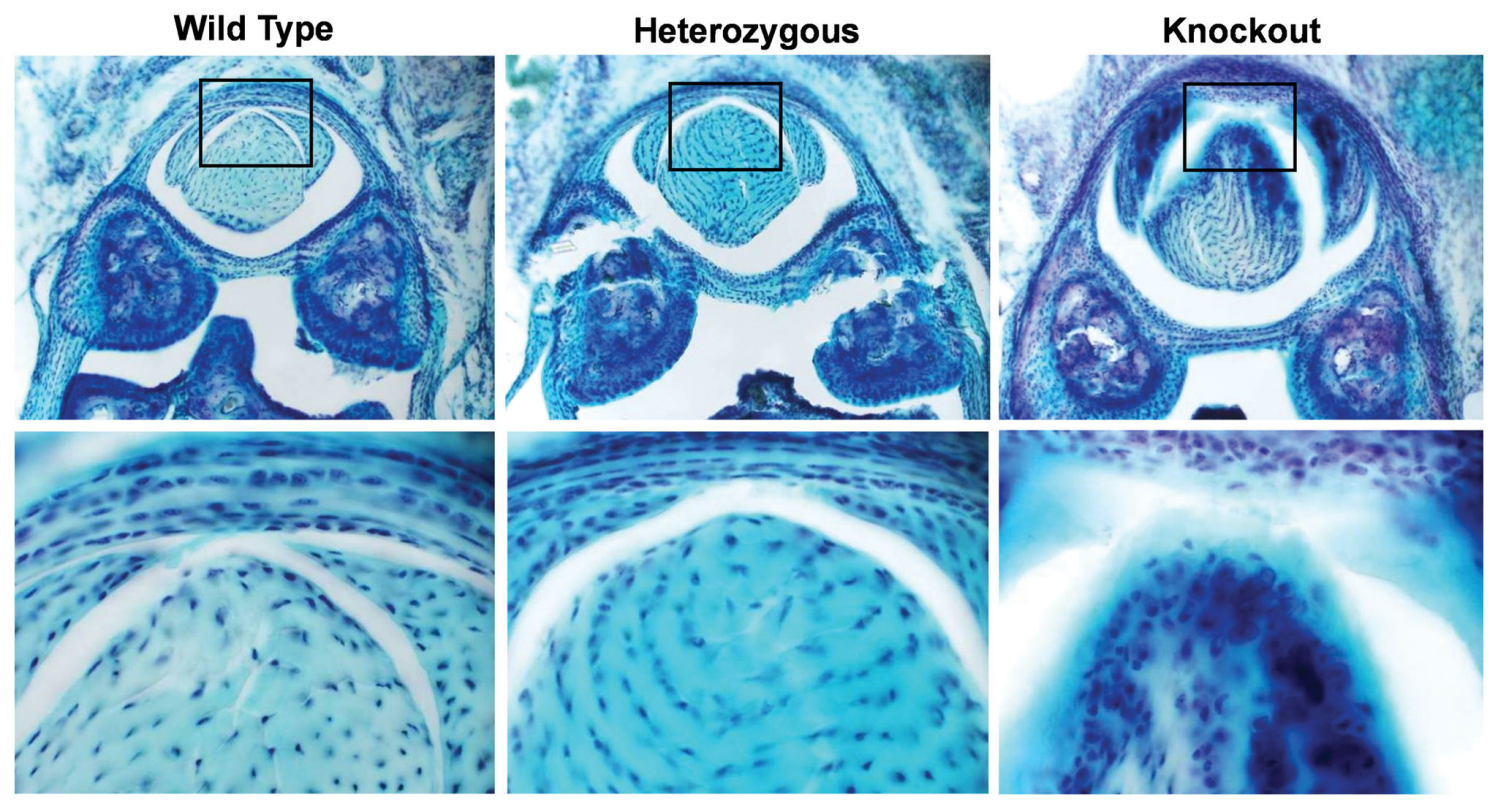
Alcian blue stain. Upper and lower row shows lower (x100) and higher (x400) magnification of the cross sections of the mouse digit respectively. Chondrocyte like formation were found in the knockout tendon.

On hematoxylin and eosin staining, the surface of the lubricin knockout tendons appeared more cellular compared to the tendons from wild type and lubricin heterozygous litter mates. Apparent cellularity was similar in wild type and heterozygous tendons. The proximal pulley appeared to be qualitatively thicker in lubricin knockout mice than in the wild type and lubricin heterozygous litter mates. (Figure 5) However, the proximal pulley in the lubricin heterozygous mice was not completely normal, as it also appeared to be somewhat thicker compared to the wild type litter mates. Despite variations in proximal pulley thickness, cellular architecture was normal in appearance in the wildtype and lubricin heterozygous mice, but lubricin knockout mice showed hyperplasia, particularly at and beneath the bearing surface of the pulley. These areas and the adjacent tendon revealed a cartilage-like tissue in the lubricin knockout mice. Tenosynovial hyperplasia was observed in the lubricin knockout mice tendon sheaths, which also appeared thickened. (Figure 6) Rounded cells, similar in appearance to chondrocytes, were seen in this tissue, and around these cells the pericellular matrix stained positive with Alcian blue. (Figure 7) Alcian blue staining was not seen in the tendons or pulleys of the wild type or heterozygous mice. 

## Discussion

The role of lubricin in the boundary lubrication of articular cartilage has been studied in detail[4–7]. Lack of lubricin in synovial fluid results in proteinaceous deposit on the cartilage surface and causes surface irregularities[7]. In contrast, the role of lubricin in intrasynovial tendon function has been less well elucidated[19]. This study was motivated by the need to better understand the role of lubricin in intrasynovial tendon, which may be useful in determining the effect of lubricin on tendon injury and subsequent operative repair in restoring normal gliding.

In this study, we developed a new system that is able to precisely assess gliding resistance in small tendons. Using this system we found that the DDF gliding resistance increased in knockout mice compared to wild type and heterozygous mice. This result supports previous reports, which examined the effect of lubricin surface modification on extrasynovial or intrasynovial tendon gliding resistance[13,14]. In these studies tendon surface modification using lubricin and carbodiimide derived gelatin with hyaluronic acid (cd-HA-gelatin) improved tendon gliding. Conversely, Sun et al. demonstrated that removing tendon surface lubricin by enzymatic digestion increased tendon gliding resistance[19]. Although these studies did not measure the gliding resistance of tendon in vivo, the results support an important role for lubricin in the maintenance of low tendon gliding resistance. 

In this study, we found some histological and structural changes in the lubricin knockout tendons. Rhee et al reported synovial hyperplasia in the knee joint of the lubricin knockout mouse[7]. They also found that lubricin inhibited synovial cell growth in vitro. In our study we did not assess the effect that purified lubricin may have on the growth of synovial cells isolated from tendon sheath, though the same mechanism probably occurs in the tendon sheath as well. 

We noted what appeared to be chondroid metaplasia in the knockout tendons. The matrix in the area was positively stained with Alcian blue and we observed a somewhat more rounded cell morphology, similar to that seen in chondroid tissue and different from that seen in tendon or ligament. Interestingly, chondroid metaplasia has also been found in the A1 pulley in adult trigger fingers[20,21]. Ehlers et al. reported that fibroblasts from tendon change their shape to a chondrocytic morphology and produce cartilage-like matrix[22]. They also reported that mechanical compression induces tendon fibrocartilage[23,24]. It is possible that the increased gliding resistance present in the knockout tendons may contribute to the induction of chondroid metaplasia. Recently, stem/progenitor cells have been identified in tendons[25]. These cells have universal characteristics of stem cells characteristics including clonogenicity, multipotency and self-renewal. These stem/progenitor cells may be the source of the chondroid-metaplasia seen in our lubricin knockout mouse tendons. 

Based on our observations here, we believe that variations in lubricin and lubrication are important for maintenance of the integrity of tendon and pulley surfaces. Clinically, these results are consistent with the possibility that a decrease in lubricin production or an alteration in the post-translational modification of lubricin in adult tissue[26] might be one of the pathogenic mechanisms, for example, of trigger finger. They also suggest that the camptodactyly in CACP syndrome might be due, at least in part, to tendinopathy, altered tendon lubrication, and the development of pathology similar to that seen in degenerative conditions such as adult trigger finger. Kostrominova et al. have reported that the expresion of lubricin and elastin is decreased in the tendons of aged rats (aged 22-25 months)[27]. They speculated that these changes may be associated with an increased risk of tendon injury with aging. Thus the lubricin knockout condition might induce a kind of ‘premature aging’ of tendons. It would be interesting to identify the developmental stage at which these changes first become evident in lubricin knockout mice, and we plan such studies in the future.

SEM revealed that the surface of lubricin knockout mouse tendon appeared rougher compared to wild type and heterozygous mice. These results are similar to those seen in extraarticular tendons, which are naturally deficient in surface lubricin[13,14]. Of clinical relevance, when lubricin is synthetically bound to the surface of such tendons, the surfaces are protected from abrasion during cycles of repetitive motion[13,14,28]. Drewniak et al.[29], showed that in heterozygous lubricin knockout mice, friction is initially normal, but it gradually increases with repetitive motion, suggesting that not only the presence but also the amount of lubricin is important. One lubricin allele may not be sufficient to protect a heavily loaded joint. Based on our observations here, we believe that lubricin treatment might be useful in both trigger finger and in treating the camptodactyly of CACP syndrome, and is deserving of further investigation.

One limitation of this method for measuring gliding resistance is the necessary assumption that the tension generated by the 5-g weight is constant. Testing of tendon gliding resistance usually includes a force transducer attached to both the proximal and distal ends of the tendon. Due to the small size of the specimen, attaching ring transducers in line with the tendon would add significant bulk to the test set-up. Since the tension caused by the 5-g weight will vary only when accelerating, and since periods of acceleration are short and of low magnitude, this assumption should not add significant error to the measurements. 

A second limitation of the study was that we did not measure the other parameters that may be affected by lack of lubricin, such as mechanical properties of the tendon, gene expression in the tendon and surrounded tissues and a more detailed characterization of the chondroid appearing cells. We also did not measure the expression of cytokines, to assess for the presence of any inflammatory reaction. Such studies may provide clues not only to the pathology in CACP syndrome but also to the underlying cause of age related tendon pathology such as trigger finger, chronic tendinopathy, subcutaneous rupture of tendon and rotator cuff tear, which occur in locations where friction between a tendon and adjacent structures is a prominent mechanical phenomenon. 

A third limitation was that we did not assess the effect of adding lubricin to the surface of the knockout tendons, to see if tendon lubrication could recover with this treatment. Finally, a fourth limitation was that the surface morphological and histological evaluations by SEM and H&E staining were not quantitative, although direct observations seemed to show obvious differences. 

In this study, we did not assess the effect of the lubricin knockout condition on tendon healing. Wong et al. have examined the mouse hind paw anatomy and developed a mouse model of flexor tendon injury[17,30]. Using this model in normal and lubricin deficient mice, we may be able to identify the effect of lubricin on not only tendon healing but also adhesion formation.

In conclusion, we present evidence to support the hypothesis that lubricin plays an important role in intrasynovial tendon gliding. To perform this study, we developed a new system that is able to assess gliding resistance between the intrasynovial tendon and apposing pulley in a mouse. A lack of lubricin is associated with poorer tendon gliding function. The lubricin knockout mouse may be useful in determining the effect of lubricin on tendon healing and the response to injury.
